# How Eyelid Changes May Impact on Tears

**DOI:** 10.3390/jcm13226927

**Published:** 2024-11-18

**Authors:** Antonio Di Zazzo, Edoardo Villani, Stefano Barabino, Giuseppe Giannaccare

**Affiliations:** 1 Ophthalmology Operative Complex Unit, University Campus Bio-Medico, 00128 Rome, Italy; 2 Eye Clinic, San Giuseppe Hospital, Istituto di Ricovero e Cura a Carattere Scientifico (IRCCS) Multimedica, University of Milan, 20123 Milan, Italy; edoardo.villani@unimi.it; 3 Ocular Surface & Dry Eye Center, Azienda Socio-Sanitaria Territoriale (ASST) Fatebenefratelli Sacco, Università di Milano, 20122 Milan, Italy; stefano.barabino@unimi.it; 4 Eye Clinic, Department of Surgical Sciences, University of Cagliari, 09124 Cagliari, Italy; giuseppe.giannaccare@unicz.it

**Keywords:** dry eye disease, meibomian gland dysfunction, blepharitis

## Abstract

This article examines the impact of eyelid margin diseases on tear film composition and associated ocular surface disorders. It highlights the prevalence of blepharitis and meibomian gland dysfunction, discussing risk factors and diagnostic considerations. Various therapeutic approaches, including eyelid hygiene, antibiotics, and innovative treatments, are explored. Emphasizing the chronic nature of these conditions, the article underscores the need for patient compliance. Overall, it provides a concise overview of eyelid-related issues and potential management strategies.

## 1. Introduction

### 1.1. Role of the Eyelids in Dry Eye Disease (DED)

The eyelids play a crucial role in the ocular surface system, directly influencing tear film regeneration, stability, and clearance, while also protecting the ocular surface and indirectly supporting neuroendocrine–immune functions mediated by the tear film [1]. Alterations in the eyelids can result in ocular surface system failure, potentially leading to severe dry eye disease (DED). Palpebral dysfunction refers to abnormalities in eyelid function and structure, which can cause two primary types of DED: evaporative and toxic. Evaporative DED arises from conditions such as deficient blinking, malocclusion syndrome, or marginal abnormalities, all of which interfere with the normal distribution of tears over the ocular surface, resulting in dryness. Conversely, toxic DED is caused by inadequate tear clearance, leading to the buildup of metabolites [2] and neuromediators [3] that can harm the ocular surface. This can occur due to deficits in the palpebral pump or obstructive outflow resulting from chronic conjunctival diseases that cause scarring. Although lid margin diseases, such as blepharitis, are not typically sight-threatening, they can lead to permanent damage, including vision loss due to superficial keratopathy, corneal neovascularization, or ulceration [4].

### 1.2. Epidemiology and Classification of Blepharitis

Blepharitis, defined as eyelid inflammation, is among the most common ocular conditions, affecting 37% to 47% of patients and occurring in both children and adults [5,6]. Acute blepharitis, which can be caused by bacterial, viral, or parasitic infections, is rare, with the chronic form being more common. Chronic blepharitis is typically categorized into staphylococcal, seborrheic, and meibomian gland dysfunction (MGD)-related forms. Anterior blepharitis involves inflammation at the base of the eyelashes and follicles (staphylococcal and seborrheic types), while posterior blepharitis affects the posterior lid margin (associated with MGD), and marginal blepharitis involves both the anterior and the posterior lid segments [7,8,9,10]. Variants of coagulase-negative Staphylococcus aureus have been detected on the eyelids of patients diagnosed with blepharitis [11]. Only 8% of healthy individuals without blepharitis had cultures positive for Staphylococcus aureus, compared to 46% to 51% of patients with staphylococcal blepharitis [12,13]. In such cases, toxin production [14] or staphylococcal antigens may trigger an inflammatory response [15,16,17,18,19]. Patients with seborrheic blepharitis exhibit greasy scaling of the anterior eyelid often associated with seborrheic dermatitis of the scalp and eyebrows.

### 1.3. Pathophysiology of MGD

**Figure 1:**
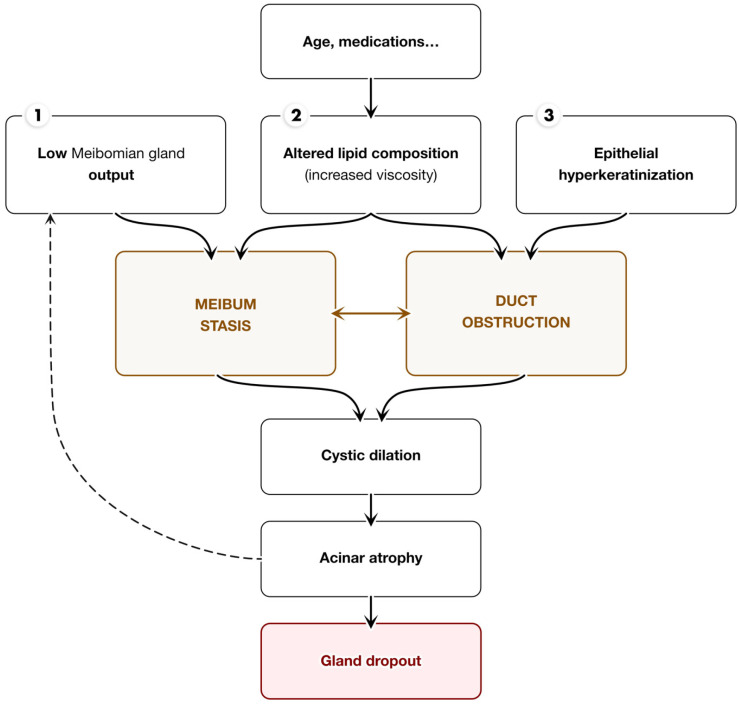
Pathological mechanisms underlying Meibomian Gland Dysfunction.

Meibomian gland dysfunction (MGD), the leading cause of evaporative DED, is one of the most common conditions encountered in daily ophthalmology practice. MGD is primarily caused by obstruction of the meibomian gland ducts and/or alterations in glandular secretion, resulting in tear film instability, inflammation, and symptoms of irritation and dryness. Increased concentrations of the sphingolipid metabolite ceramide in meibum raise the meibum melting temperature, destabilizing the outer, non-polar tear film lipid layer [20,21]. Meibomian gland dysfunction (MGD) involves a series of molecular events that drive hyperkeratinization and ductal obstruction, impairing lipid secretion. Pro-inflammatory cytokines, such as IL-1β, IL-6, and TNF-α, are elevated in MGD, promoting keratinocyte proliferation and turnover, which contributes to the accumulation of keratinized cells in gland ducts. Matrix metalloproteinase-9 (MMP-9) further exacerbates this process by activating IL-1β and degrading extracellular matrix components, leading to glandular blockage. Additionally, keratins, particularly keratin-1 and keratin-10, are overexpressed in the ductal epithelium, contributing to obstruction and reduced tear film stability. These combined factors initiate a cycle of inflammation and obstruction that compromises tear film integrity and exacerbates dry eye symptoms. Both hyposecretory (low-delivery) and hypersecretory (high-delivery) MGD are linked to either insufficient secretion or gland obstruction [22,23,24] (Figure 1).

This article aims to underscore the significance of the eyelids in the ocular surface system, providing a comprehensive overview of eyelid-related conditions that contribute to dry eye syndrome. The various causes of blepharitis and meibomian gland dysfunction, as well as related complications, are discussed with the overall objective of improving patients’ quality of life by restoring tear film stability and promoting proper eyelid gland function.

## 2. Methods

The methodology employed in this scientific review involved a comprehensive search and analysis of relevant literature to investigate the various aspects of eyelid alterations and their association with dry eye disease (DED). The review aimed to provide insights into the mechanisms, risk factors, clinical manifestations, and therapeutic approaches related to palpebral dysfunction. To conduct the literature review, multiple electronic databases (such as PubMed, Google Scholar, and ResearchGate) and academic repositories (such as arxiv and SSRN) were systematically searched using specific keywords related to eyelid alterations, dry eye disease, risk factors, symptoms, clinical signs, and therapeutic interventions. The search strategy included peer-reviewed articles, clinical trials, systematic reviews, and meta-analyses published in scientific journals from 2010 to 2024 to better unveil critical developments in modern dry eye disease. The types of published studies reviewed encompassed a wide range of research methodologies, including experimental studies, observational studies, clinical trials, case–control studies, and case series. These studies were selected based on their relevance to the topic and their contributions to understanding the pathophysiology, diagnosis, and management of eyelid alterations and associated dry eye disease. The review process involved screening the identified studies based on predefined inclusion and exclusion criteria to ensure the selection of high-quality and pertinent literature. Data extraction was performed to extract relevant information, including study objectives, methodologies, results, and conclusions. The extracted data were then synthesized and analyzed to identify key findings, trends, and gaps in the existing literature. Furthermore, the review included a critical appraisal of the selected studies to assess their methodological quality, risk of bias, and generalizability of findings. The strengths and limitations of individual studies were carefully considered to provide a balanced interpretation of the evidence. Overall, the methodology employed in this scientific review aimed to systematically gather, evaluate, and synthesize the available evidence to offer a comprehensive understanding of eyelid alterations and their implications for ocular surface health. By synthesizing findings from diverse sources, this review contributes to advancing knowledge in the field and guiding clinical practice and future research endeavors.

## 3. Risk Factors

Eyelid margin diseases, resulting from tear film dysfunction, encompass a variety of conditions characterized by inflammation, irritation, and structural changes along the eyelid margins. These conditions include:

1. Blepharitis: Blepharitis is a chronic inflammatory disorder affecting the eyelid margins frequently caused by bacterial overgrowth, infestation by Demodex mites, or meibomian gland dysfunction. It often presents with symptoms such as itching, burning, and crusting along the eyelid margins.
2. Meibomian gland dysfunction (MGD): MGD is a prevalent disorder in which the meibomian glands, responsible for producing the lipid component of the tear film, are impaired. This dysfunction results in poor quality or insufficient meibum, leading to evaporative dry eye and inflammation of the eyelid margins.
3. Marginal keratitis: Marginal keratitis involves inflammation of the peripheral cornea typically caused by chronic irritation from lid margin abnormalities or compromised tear film quality. It manifests as corneal infiltrates and ulcers often accompanied by discomfort and redness.
4. Trichiasis: Trichiasis is a condition where eyelashes grow inward toward the ocular surface, causing constant irritation of the cornea and conjunctiva, which can lead to inflammation and potential ocular surface damage.
5. Distichiasis: Distichiasis is a congenital or acquired condition characterized by the presence of abnormal eyelashes emerging from the meibomian gland orifices. These extra lashes can irritate the ocular surface, causing symptoms such as a foreign body sensation and excessive tearing.
6. Chalazion: A chalazion is a localized swelling on the eyelid caused by the obstruction and inflammation of a meibomian gland. It typically presents as a firm, painless nodule along the eyelid margin sometimes accompanied by redness and tenderness.

Risk factors for eyelid margin diseases include advancing age, poor eyelid hygiene, ocular surface conditions such as dry eye disease and rosacea, contact lens use, environmental exposure to pollutants and allergens, systemic diseases such as diabetes and autoimmune disorders, and the use of certain medications like isotretinoin and topical steroids [4,25,26,27,28].

Several factors contribute to tear film alterations and ocular surface dysfunction associated with eyelid margin diseases. Aging is a well-established risk factor for MGD as meibomian gland acinar epithelial cells undergo atrophy, lipid production decreases [29], and alterations in meibum composition, involving changes in neutral and polar lipids, become more prevalent [25,30]. Aged meibomian glands exhibit reduced meibocyte differentiation, renewal, and size, along with increased immune cell infiltration [31,32,33,34,35,36].

The ocular surface microbiome, including Staphylococcus aureus at the eyelid margin, may be influenced by meibum cholesterol esters. Commensal bacteria, through their lipolytic enzymes, break down neutral fats and esters, releasing glycerides and free fatty acids (polar lipids) into the tear film, thereby altering meibum composition [22]. A diet rich in omega-3 fatty acids has been shown to improve meibum quality and expressibility in such cases [37,38].

Congenital absence of meibomian glands has been observed in conditions like Turner syndrome and ectrodactyly–ectodermal dysplasia–cleft lip/palate (ECC syndrome), which are associated with severe tear film instability [39]. Environmental stress, particularly low humidity, also contributes to MGD by exacerbating tear film instability, as demonstrated in both human and animal models of severe dry eye disease [40].

Hormonal changes and systemic hormone therapies, such as estrogen replacement in women and anti-androgen treatment in men, also influence the development of MGD and blepharitis [41,42,43]. Androgens generally promote lipid secretion by stimulating lipogenesis and suppressing keratinization-related genes, while also enhancing acinar cell maturation. Androgen deficiency, receptor dysfunction, or anti-androgen treatments may contribute to obstructive MGD. In contrast, estrogens, particularly 17-β-estradiol, tend to inhibit lipogenesis, reducing lipid output from sebaceous glands, including the meibomian glands, which negatively affects lipid secretion and promotes inflammation on the ocular surface [44].

Several secondary causes, such as ocular mucous membrane pemphigoid (OMMP), Stevens–Johnson syndrome/toxic epidermal necrolysis, connective tissue diseases, discoid lupus, dermatomyositis, graft-versus-host disease (GVHD), pemphigus foliaceus, erythema multiforme minor/major, atopic dermatitis, and psoriasis, can contribute to anterior blepharitis and cicatricial MGD. Seborrheic dermatitis and acne rosacea are known secondary causes of both obstructive non-cicatricial and hypersecretory MGD [7]. Low meibomian gland output and epithelial hyperkeratinization result in duct obstruction, meibum stasis, cystic dilation, acinar atrophy, and eventual gland dropout [44,45,46].

Other ocular factors associated with MGD include conditions like aniridia, eyelid tattooing, contact lens use, ocular surgeries, floppy eyelid syndrome, giant papillary conjunctivitis, and trachoma [41]. Topical glaucoma medications, including beta-blockers, prostaglandin analogs, and carbonic anhydrase inhibitors, are associated with morphological changes in the meibomian glands, such as reduced acinar area, acinar density, and homogenous acinar wall structure [47,48]. Systemic medications, such as 13-cis-retinoic acid, have been linked to severe atrophy of the meibomian glands [49], while isotretinoin, an oral treatment for severe acne, has been shown to increase Staphylococcus aureus colonization, leading to anterior blepharitis [49,50,51,52].

Finally, infestation by Demodex mites, particularly Demodex folliculorum in lash follicles and Demodex brevis in the sebaceous and meibomian glands, has been implicated in 90% of anterior blepharitis cases and 60% of MGD cases [53]. Demodex mites cause direct damage to hair follicles, leading to reactive hyperkeratinization and cylindrical dandruff formation. D. brevis obstructs the meibomian glands through granulomatous reactions, predisposing affected individuals to MGD and chalazia [54].

## 4. Ocular Surface Symptoms and Clinical Signs

Blepharitis is a frequent cause of ocular surface inflammation across all age groups and ethnicities, manifesting as conditions ranging from conjunctivitis and functional tear deficiency to keratitis. It may also exacerbate the symptoms of pre-existing ocular surface diseases, such as aqueous tear deficiency and allergic reactions [55]. Common symptoms include burning, irritation, a gritty sensation, intolerance to contact lenses, photophobia, as well as redness and crusting at the eyelid margins. These symptoms tend to be worse in the morning and fluctuate throughout the day with episodes of exacerbation and remission. In severe cases, blepharitis can lead to permanent alterations of the eyelid margin or cause vision loss due to superficial keratopathy, corneal neovascularization, or ulceration.

Staphylococcal (anterior) blepharitis is often characterized by erythema and edema at the eyelid margins and accompanied by telangiectasia. Patients may also present with brittle scales around the eyelashes that form collarettes, encircling the lash at the base or along the shaft as the lash grows. In advanced or chronic cases, trichiasis (inward-growing eyelashes), poliosis (loss of eyelash pigmentation), madarosis (loss of eyelashes), eyelid ulceration, and scarring of the eyelid and cornea may occur—symptoms less frequently observed in other types of blepharitis. Additionally, hordeolum, an inflammatory nodule of the eyelid arising from the hair follicles or meibomian glands, is a common complication of this blepharitis type.

Seborrheic blepharitis is frequently associated with seborrheic dermatitis, characterized by oily or greasy eyelid deposits, mild conjunctival redness, and punctate epithelial erosions in the lower cornea [56]. In contrast, Demodex blepharitis is linked to ocular rosacea and age over 70, with characteristic signs such as a “sleeve” or scurf encircling the base of the lashes, significant debris, irregular eyelid margins, and madarosis [27,45]. Notably, the severity of ocular surface discomfort shows a strong positive correlation with the number of Demodex mites per lash [57].

Posterior blepharitis is diagnosed by examining the posterior eyelid margin, where the meibomian glands often appear capped with oil, dilated, or obstructed. The gland secretions are typically thickened, sometimes taking on a toothpaste-like consistency. As with other forms of blepharitis, telangiectasia and scarring of the eyelids may be observed.

The prevalence of MGD increases with age [58], occurring equally in men and women [13], but it is more common among individuals with fair skin, especially those with acne rosacea [58], and in colder climates [59]. Acne rosacea is co-diagnosed in approximately 20% of MGD cases [11,60], while 46% of MGD patients also present with seborrheic dermatitis [11]. Chalazia are a frequent complication in individuals with MGD.

MGD patients show greater fluorescein staining of the cornea compared to those with normal meibum, likely due to increased tear evaporation, which leads to surface hyperosmolarity and a compensatory reflex increase in aqueous tear production [61,62]. The lipid component of the tear film can be indirectly assessed through measurements of tear osmolarity, tear break-up time, oil imprinting, and lipid layer thickness [63,64]. Terminal duct obstruction due to hyperkeratinization and increased meibum viscosity can result in gland dropout, atrophy, and reduced secretion.

The functional assessment of MGD focuses on the effects of lipid quantity and quality on tear film stability. In contrast, imaging techniques such as transillumination [65,66,67], non-contact infrared (IR) light imaging [68], confocal microscopy [69,70,71], and optical coherence tomography (OCT) [72] provide morphological evaluations of the meibomian glands [73,74]. Transillumination using a contact plate or probe-type illuminator enables detailed visualization of meibomian gland structure with high image quality. IR transillumination, confocal microscopy, and OCT have been introduced to more precisely visualize meibomian gland structure [72,75].

In vivo 3D imaging techniques, including in vivo confocal microscopy (IVCM), optical coherence tomography, and keratography, allow for the acquisition of depth information and enhanced visibility of the meibomian glands [72]. In addition, the ocular surface analyzer has emerged as a valuable tool, allowing for detailed assessment of both meibomian gland structure and tear meniscus height, which is critical for evaluating tear dynamics and ocular surface health. Recent studies have highlighted its utility in comprehensive ocular surface analysis, offering insights that complement those from other imaging modalities [76].

## 5. Therapy

Blepharitis and related diseases associated with lid margin dry eye are typically chronic and challenging to treat. The primary goal of therapy is to enhance the patient’s quality of life by improving gland secretion flow and re-establishing tear film stability [77]. Effective non-invasive treatment options, such as eyelid hygiene and warm compresses, have been used for years. For patients with MGD, warming the eyelids, followed by cleansing and gentle massage to express the meibomian glands, is considered a fundamental part of the therapy. Devices like MGDRx, Eyebag (The Eyebag Company, West Yorkshire, UK), Blephasteam (Laboratoires Théa, Clermont-Ferrand, France), and LipiFlow (TearScience, North Carolina, USA) have demonstrated success in alleviating symptoms for MGD sufferers [78,79,80,81].

The International Workshop on Meibomian Gland Dysfunction (IWMGD) highlights terminal duct obstruction as a critical feature of MGD, making mechanical methods for duct opening and meibum expression vital in treatment. Techniques such as intraductal probing and the use of electronic heating devices facilitate the mechanical opening of the duct orifice and/or heating of the meibum to improve outflow.

The melting point of meibomian lipids ranges between 19 °C and 32 °C [82], allowing meibum to remain fluid under normal conditions [21]. However, in MGD, alterations in meibum composition increase the melting point, leading to more frequent obstructions [83,84]. By raising the eyelid temperature, meibomian lipid viscosity is reduced, improving lipid levels along the lid margin [21]. In more severe cases of MGD, temperatures exceeding 40 °C are needed to liquefy obstructive material within the glands [83].

For managing staphylococcal and seborrheic blepharitis, topical antibiotics—particularly in ointment form—are recommended. These should be applied following eyelid hygiene procedures, once or twice daily, depending on the severity of inflammation [85]. Commonly prescribed antibiotics include erythromycin, doxycycline, and bacitracin. Treatment is typically discontinued within two to eight weeks or once symptoms have resolved, although in rare cases, chronic use may be necessary [13].

Antibiotics such as tetracyclines and azithromycin are known to effectively reduce eyelid inflammation [29]. Bacteria produce toxic metabolites, enzymes (e.g., lipases), and pro-inflammatory molecules like matrix metalloproteinases (MMPs), all of which damage the ocular surface. The presence of commensal bacteria on the eyelid margins, which is elevated in blepharitis patients compared to healthy individuals, supports the longstanding use of antibiotics in MGD treatment [86].

Tetracyclines work by inhibiting the local production of lipases and MMPs, as well as the bacterial release of pro-inflammatory molecules like free fatty acids, which contribute to tear film instability and inflammation at the meibomian glands [87,88,89,90]. These drugs also modulate neutrophil and lymphocyte activity and exhibit antioxidative properties. Lipophilic tetracyclines, such as doxycycline and minocycline, achieve higher concentrations in ocular and lid tissues at lower doses (50–100 mg once or twice daily) compared to other tetracyclines, which may require higher doses (250–300 mg up to four times daily) [67,91].

Azithromycin not only reduces bacterial growth on the eyelid margins but also stimulates phospholipidosis, suppresses bacterial lipases, and decreases conjunctival inflammation [92,93,94]. Studies have shown that topical azithromycin (1.5%), when used for a longer course of one month (versus three days), alongside daily lid hygiene, significantly improves meibomian gland function. Oral azithromycin has also been found effective, with a single 1 g dose maintaining high concentrations in conjunctival tissue and tear fluid for at least 14 days [95,96]. While adverse cardiac events are rare, caution is advised.

Treating Demodex infestations can significantly improve blepharitis and reduce eyelid inflammation. Tea tree oil (TTO), particularly terpinen-4-ol, its active ingredient, has been shown to effectively eliminate Demodex and alleviate inflammation associated with the mites [97,98,99]. A one-month regimen of 10% lid scrubs eradicates Demodex and reduces the related inflammation [57,99]. Given the potential side effects, such as contact dermatitis, 22terpinen-4-ol is recommended for topical use over the lids for mild to severe symptoms, with a trial period of two months [93,97].

For more resistant cases of Demodex blepharitis, oral ivermectin can be used to reduce the number of mites and improve symptoms [100,101,102]. Hormonal therapy remains an area of active research in the treatment of MGD, with some evidence supporting the use of 5% testosterone cream to improve meibomian gland secretion in peri- and postmenopausal women. Additionally, a randomized controlled trial found that topical 0.03% testosterone ophthalmic solution significantly improved meibomian secretion viscosity over six months [96].

Although the exact role of inflammation in MGD pathophysiology is still unclear, elevated levels of phospholipase A2 in the meibum of patients with blepharitis suggest its involvement in the synthesis of inflammatory mediators like prostaglandins and leukotrienes. Cytokines, such as IL-1α, further exacerbate epithelial proliferation and keratinization, contributing to MGD [103,104].

Topical corticosteroids, including loteprednol etabonate and fluorometholone, may help control inflammation, particularly in severe cases involving conjunctival inflammation, marginal keratitis, or phlyctenules. After controlling the inflammation, corticosteroids can be tapered and discontinued, though intermittent use may be necessary for patient comfort during acute inflammatory episodes [105]. The combination of a topical antibiotic and corticosteroid is beneficial for managing blepharitis since bacterial infection and inflammation often coexist. While short-term use of these combinations has been shown to provide mild benefits, treatment durations rarely exceed 30 days.

Topical cyclosporine A (0.05%) (CSA) and Lifitegrast may be beneficial for select patients with posterior blepharitis. CSA is also effective in increasing aqueous production in dry eye disease and plays a role in managing inflammation in MGD, leading to improvements in lid margin redness, meibomian gland inclusions, telangiectasia, and corneal staining [106]. Studies have investigated higher concentrations of cyclosporine A, such as 0.1%, 0.2%, and 0.4%, in dose-ranging trials for the treatment of dry eye disease. However, no clear dose–response relationship was observed. Both the 0.05% and 0.1% concentrations produced significant improvements in symptoms and ocular surface staining, with 0.05% demonstrating the most consistent clinical benefits [107].

Omega-3 supplementation may further enhance meibum properties and alleviate MGD symptoms due to its anti-inflammatory effects [82,108,109,110,111].

Intense pulsed light (IPL) therapy, originally used to treat systemic rosacea [112], has been proposed as a treatment for chronic blepharitis due to its ability to reduce abnormal blood vessels and inflammatory mediators [113,114]. IPL therapy has also shown potential in improving meibomian gland functionality and tear film stability. Although promising, further studies are needed to clarify the optimal patient selection, treatment protocols, and long-term efficacy of IPL for MGD [115,116,117,118,119]. Recent evidence suggests that IPL may have a beneficial effect in managing Demodex blepharitis. The heat generated by IPL reaches the temperature required to destroy the mites and induces coagulation necrosis in the Demodex organism. This eradication can indirectly reduce the bacterial load on the eyelid, decrease the immune response, and alleviate symptoms associated with the eyelid margin and ocular surface. Since Demodex mites carry bacteria that can damage the cells of the ocular surface, especially meibomian gland cells, eliminating them can reduce the release of toxic bacterial substances, which otherwise increase the viscosity of palpebral sebum and cause further damage. While these findings are promising, further research is needed to establish the long-term efficacy of IPL in this specific condition [120].

**Figure 2:**
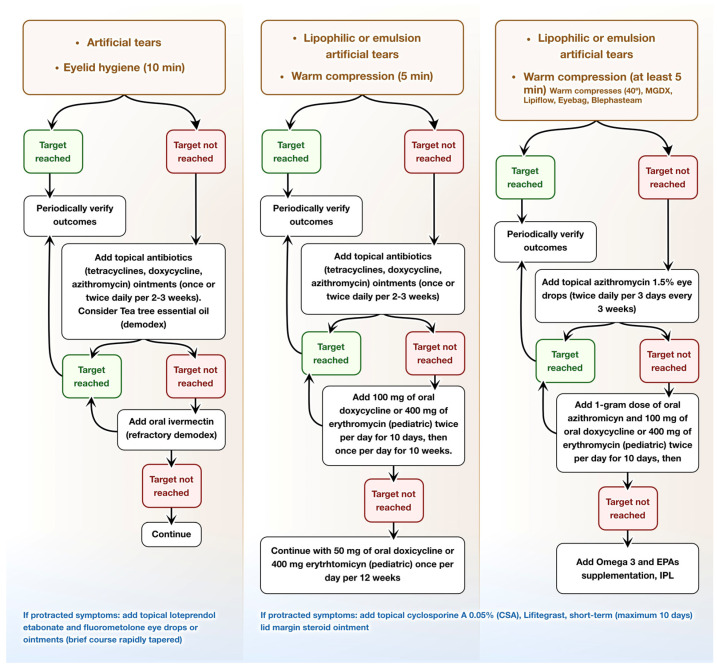
Therapeutic Approach for Meibomian Gland Dysfunction (MGD. This flowchart presents stepwise treatment pathways based on patient response to therapy.

**Figure 3:**
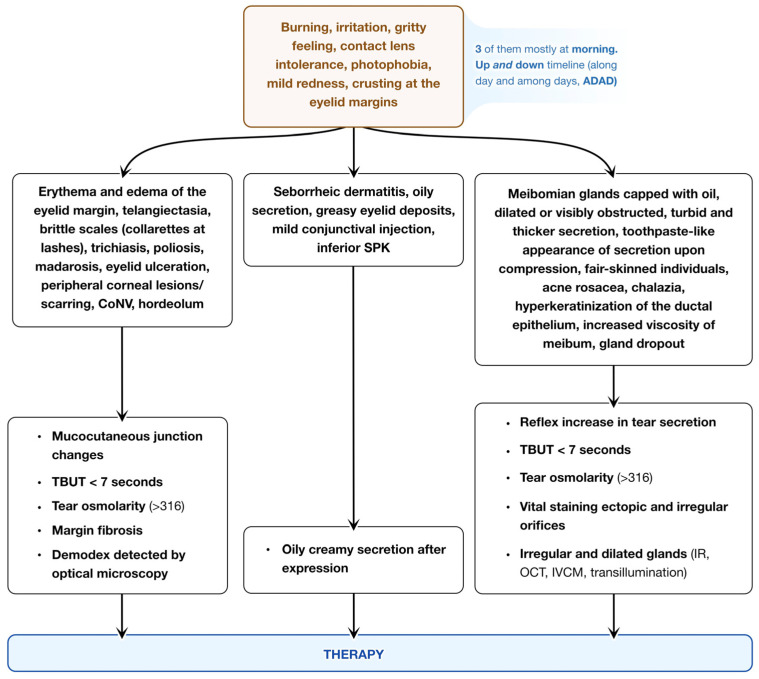
Diagnostic and Clinical Features of Meibomian Gland Dysfunction (MGD). This flowchart illustrates the clinical manifestations and diagnostic criteria associated with Meibomian Gland Dysfunction. CoNV: corneal neovascularization, SPK: superficial punctate keratitis, TBUT: tear break-up time, IR: infrared meibography, OCT: optical coherence tomography, IVCM: in vivo confocal microscopy.

Finally, intranasal tear neurostimulation has been found to enhance tear secretion, lipid production, and meibum secretion from meibomian glands by stimulating the nasolacrimal reflex. In experimental models, three minutes of daily pulsed stimulation over a period of three weeks led to increases in tear volume and lipid content, as well as reduced tear osmolarity [121,122]. The diagrams below (Figure 2 and Figure 3) outline the approach to transitioning from clinical diagnosis to treatment based on the underlying eyelid pathology.

## 6. Conclusions

Blepharitis is generally a chronic condition that lacks a permanent cure, and its effective management heavily relies on the patient’s adherence to the treatment regimen. This makes achieving satisfactory control of the condition challenging. Currently, there is no consensus on a standard treatment protocol, and there are no FDA-approved medications specifically designed for blepharitis. However, various treatment approaches have been shown to alleviate symptoms to some extent.

A combination of eyelid hygiene and antibiotic or corticosteroid therapy has proven to be a safe and effective strategy for managing blepharitis. Several studies have shown positive clinical outcomes and a strong safety profile with the use of topical azithromycin, particularly in cases of anterior blepharitis. In such cases, topical antibiotics have been effective not only in providing symptomatic relief but also in eradicating bacteria from the eyelid margins.

Oral doxycycline has also been found to contribute to clinical improvements at both high (200 mg twice daily) and low (20 mg twice daily) doses. Additionally, castor oil-based eyedrops have shown superior results compared to saline drops in enhancing tear function, particularly in terms of tear stability.

In conclusion, patients should be advised that while symptoms can often be significantly improved, complete elimination of the condition is rare.
